# A Review of Influencing Factors on Spatial Spread of COVID-19 Based on Geographical Perspective

**DOI:** 10.3390/ijerph182212182

**Published:** 2021-11-20

**Authors:** Zhixiang Xie, Rongqin Zhao, Minglei Ding, Zhiqiang Zhang

**Affiliations:** 1 College of Surveying and Geo-Informatics, North China University of Water Resources and Electric Power, Zhengzhou 450046, China; zhixiang1108@163.com (Z.X.); dingminglei@ncwu.edu.cn (M.D.); zhangzhiqiang.8866@163.com (Z.Z.); 2 Key Research Institute of Yellow River Civilization and Sustainable Development & Collaborative Innovation Center on Yellow River Civilization of Henan Province, Henan University, Kaifeng 475001, China; 3 Key Laboratory of Geospatial Technology for Middle and Lower Yellow River Regions, Henan University, Ministry of Education, Kaifeng 475004, China

**Keywords:** COVID-19, spatial diffusion, influencing factors, geography

## Abstract

The COVID-19 outbreak is a manifestation of the contradiction between man and land. Geography plays an important role in epidemic prevention and control with its cross-sectional characteristics and spatial perspective. Based on a systematic review of previous studies, this paper summarizes the research progress on factors influencing the spatial spread of COVID-19 from the research content and method and proposes the main development direction of geography in epidemic prevention and control research in the future. Overall, current studies have explored the factors influencing the epidemic spread on different scales, including global, national, regional and urban. Research methods are mainly composed of quantitative analysis. In addition to the traditional regression analysis and correlation analysis, the spatial lag model, the spatial error model, the geographically weighted regression model and the geographic detector have been widely used. The impact of natural environment and economic and social factors on the epidemic spread is mainly reflected in temperature, humidity, wind speed, air pollutants, population movement, economic development level and medical and health facilities. In the future, new technologies, new methods and new means should be used to reveal the driving mechanism of the epidemic spread in a specific geographical space, which is refined, multi-scale and systematic, with emphasis on exploring the factors influencing the epidemic spread from the perspective of spatial and behavioral interaction, and establish a spatial database platform that combines the information of residents’ cases, the natural environment and economic society. This is of great significance to further play the role of geography in epidemic prevention and control.

## 1. Introduction

On 30 December 2019, the Wuhan Municipal Health Commission issued an urgent notice on the treatment of pneumonia of unknown cause, stating that some medical institutions have been receiving patients with pneumonia of unknown cause in recent days, and urging them to take care of the treatment and report it on time (http://wjw.wuhan.gov.cn/, accessed on 5 January 2020). On 8 January 2020, the team identified the cause as a novel coronavirus, which has a similar transmission route to the Severe Acute Respiratory Syndrome (SARS) virus. On 12 January, the World Health Organization designated the novel coronavirus as “2019-nCoV”, predicting an incubation period of 2–10 days. Then, coronavirus (hereinafter referred to as “COVID-19”) began to enter the public eye and become a global hot topic. As of 17:30 on 12 July 2021, the cumulative number of confirmed cases worldwide exceeded 186.4 million and the cumulative death toll exceeded 4.03 million (https://www.who.int/, accessed on 12 July 2021). The ravages of the epidemic not only consume the lives and health of residents, but also hinder the process of economic globalization, causing huge economic losses to countries around the world [1,2]. In addition, the mutation of the virus strain makes the incubation period of the virus shorter and the epidemic stronger, increasing the difficulty of epidemic prevention and control. In this context, sorting out the factors influencing the spatial spread of COVID-19 has become an urgent issue to prevent and defuse the epidemic risk.

Since the outbreak of COVID-19, scholars have carried out a large number of studies on the of the epidemic spread, but the focus of research based on toxicology, mathematics, environmental science, economics and geography is different due to differences in academic backgrounds. In terms of toxicology, studies are mainly carried out from the aspects of virus type, regeneration capacity and latent cycle, with the purpose of describing the characteristics and diffusion process of viruses [3]. David et al. [4] compared novel coronavirus with other viruses and believed that the sustained epidemic would pose a serious threat to global health, and proposed that sustainable development could be achieved by constructing a human–environment–animal health alliance. Liu et al. [5] used exponential growth and maximum likelihood estimation to determine the transmission dynamics of 2019-NCOV in Wuhan, revealing that the incubation period of the virus was 4.8 days, and the basic regeneration index was 2.90 (95%CI: 2.32–3.63) and 2.92 (95%CI: 2.28–3.67). In terms of mathematics, scholars employed the infectious disease models (SI, SIR, SEIR and SIRD) to predict the development trend of the epidemic based on the data of COVID-19 cases in some countries or regions, which provided a reference value for promoting epidemic prevention and the resumption of work and production [6,7]. Studies from the perspective of environmental science focus on the impact of air quality changes and environmental factors on the epidemic spread [8,9], aiming to explore the interaction mechanism between environmental factors and epidemic. From the perspective of economics, the research focus is to discuss the impact of the COVID-19 spread on enterprises, industries, regional economy and even economic globalization [1,10,11], in order to explain the harm of the pandemic to economic development and the necessity of economic recovery measures.

Although toxicology, mathematics, economics and other disciplines have made a lot of exploration in epidemic research, geography plays an important role in epidemic prevention and control by carrying out research on spatial spread, transmission path and driving mechanism of epidemic from its unique spatial perspective [12]. The relationship between man and earth is the core of geography. Every kind of natural and human element on the earth’s surface can be the study object of geography. As a rapidly spreading infectious disease that threatens human life and health, COVID-19 has universal attributes of geographical research objects [13]. Since the beginning of the 21st century, sudden public health events such as SARS, H1N1 and COVID-19 have occurred all over the world and epidemic research has attracted the attention of geographers [14]. Studies on the spatial spread of COVID-19 from a geographical perspective can be divided into macro-regional and micro-individual levels. Studies at the macro-regional level mainly focus on the global, national, regional and urban regional scales, focusing on revealing the universal laws of spatial differentiation and driving mechanism of COVID-19 [15]. Wang et al. [16] classified the epidemic in China into the stage of spread within Wuhan, rapid spread in multiple places, rapid increase in confirmed cases, decrease in the fluctuation of the increase rate of confirmed cases, control of the epidemic, and import from abroad. They believed that geographical proximity, strength of social and economic linkages, and human activities were the main factors affecting the epidemic spread. Liu et al. [17] explored the process of the epidemic spread at the county level from the total number of cases, the number of imported spread cases and the spread ratio, and found that the epidemic distribution reflected the characteristics of geographical proximity and network proximity. Fu et al. [18] believed that the outbreak and transmission of the epidemic not only came from its own toxicity and transmission capacity, but also was closely related to social, economic and environmental factors such as population flow, and proposed to build a systematic plan to fight the epidemic from the aspects of coordination, classification and collaboration. The micro-individual studies emphasize that the spatial and temporal transmission paths of the epidemic and the rules of individuation should be described from physiological differences, behavioral preferences, geographical background uncertainty and economic and social attributes. Chai et al. [14] took spatio-temporal path expression, complex scenario analysis and risk map perception as the core research methods to build the research framework of spatio-temporal behavioral geography for epidemic prevention and control, providing a reference value for the prevention and control of sudden public health events. Kumar et al. [19] focused on exploring the factors influencing the epidemic spread from the perspective of social attributes and uncertainty of geographical background, revealing that age, gender, immunity, malnutrition and family poverty are the main reasons influencing the epidemic spread. Chen et al. [20] used factor analysis and regression analysis to explore the influencing factors of the spatial spread of the epidemic in Chongqing, and found that stores, supermarkets, restaurants and other living service locations had the greatest impact on the epidemic spread. In summary, current studies mostly explore changes in virus regeneration capacity from the perspective of toxicology and mathematics, so as to determine epidemic stages and predict future development trend. Although some scholars try to reveal the law of epidemic spread from the perspective of geography, they only focus on the description of spatial-temporal characteristics of the epidemic and rarely involve the driving causes of epidemic spread [21]. In addition, the research methods and contents of the discussion on factors affecting the epidemic spread are fragmented, which weakens the application value of the evaluation results to some extent. In view of this, this paper reviews the factors influencing the spatial spread of COVID-19 from a geographical perspective and looks into the development direction of geography in epidemic prevention and control, which provides reference for future emergent public health events.

## 2. Research Contents

The causes of the spatial spread of COVID-19 are complex. To clarify the influencing factors of the spatial spread of COVID-19 and summarize the driving mechanism of epidemic spread is not only the premise for the rational allocation of epidemic prevention and control resources and scientific development of epidemic control measures, but also the path choice for an effective response to unexpected public health events and improvement of public health systems in the future [14]. At present, the academic research on the influencing factors of the spatial spread of the epidemic is generally divided into two aspects: natural environment and economic and social factors.

### 2.1. Impact of Natural Environmental Factors on the Epidemic Spread

Natural environment, also known as natural complex, refers to the organic whole composed of atmosphere, water, rocks, organisms and other derived natural substances on the surface of the Earth [22]. According to the concept of natural environment, it is not difficult to find that natural environmental factors generally refer to all non-human created basic material components with different nature that directly and indirectly affect human life and production environment, including water, atmosphere, organisms, sunlight, soil and so on. As COVID-19 spreads mainly through respiratory droplets and contact transmission, atmospheric factors play an important role in the process of the epidemic spread. Current studies on the impact of atmospheric factors on the COVID-19 spread have focused on meteorological conditions such as temperature, humidity, wind speed and rainfall, as well as sulfur dioxide (SO_2_), nitrogen oxides (NO_x_) and particulate matter (PM) and other air quality aspects [23].

In terms of meteorological conditions, Araujo and Naimi [24] from Spain and Finland used the niche model to explore the appropriate temperature for the COVID-19 spread. The results showed that the COVID-19 spread was easier in temperate and cold zones than in tropical areas, which was the beginning of the study on the impact of meteorological conditions on the COVID-19 spread. Then, scholars from the University of Maryland used a linear regression model to reveal the relationship between confirmed COVID-19 cases and temperature and latitude, and believed that the 30–50° N region has a high risk of epidemic transmission. This study again verified that meteorological factors have a significant impact on epidemic transmission [25]. After that, studies conducted by scholars on the impact of meteorological factors on the COVID-19 spread are increasing rapidly and are increasingly refined on the regional scale (from the global scale to the urban scale). On the global scale, Bariotakis et al. [26] predicted the epidemic trend based on bioclimatic variables and concluded that the region with the highest infection rate would shift from Europe to Southeast Asia. On the national scale, Zhu et al. [27] used the Spearman correlation coefficient to evaluate the relationship between meteorological factors (average temperature, maximum temperature, minimum temperature, wind speed and air humidity) and confirmed and latent cases in four South American countries, and found that air humidity was negatively correlated with the epidemic incubation period. Tosepo et al. [28] used the Spearman correlation coefficient to explore the impact of the lowest temperature, highest temperature, average temperature, humidity and rainfall on epidemic transmission in Indonesia, revealing that the average temperature was closely related to the epidemic. Gupta et al. [29] used the distribution model to explore the impact of temperature and humidity on the epidemic spread and believed that temperature and humidity had a significant impact on the epidemic spread in the United States but not in India. Qi et al. [30] used the Generalized Additive Model (GAM) to determine the correlation between meteorological variables and confirmed cases in China, and found that there was a negative correlation between temperature and humidity and confirmed cases. On the regional level, Gupta D and Gupta A [31] used regression analysis to explore the impact of temperature on epidemic transmission in California when the time lag effect was considered, and concluded that each 1 °C increase in temperature reduced the risk of epidemic transmission by 8.2%. Chien and Chen [32] used the GAM model to explore the impact of temperature, humidity and precipitation on epidemic transmission in the worst-hit areas of the United States, and believed that the increase in temperature, relative humidity and rainfall could reduce the risk of epidemic transmission. On the urban scale, Singh et al. [33] used the Pearson correlation coefficient to analyze the relationship between daily maximum high temperature, daily minimum low temperature, daily average temperature, relative humidity, sunshine duration, daily average wind speed, rainfall, evaporation and other confirmed cases in New Delhi, and found that the daily maximum temperature, daily minimum temperature, daily mean temperature, relative humidity, evaporation and daily mean wind speed were positively correlated with epidemic spread. Xie and Zhu [34] explored the relationship between temperature and confirmed cases in 122 Cities in China by using GAM and the piecewise regression model, and believed that the relationship between temperature and confirmed cases was approximately linear when the temperature was <3 °C and tended to be flat when the temperature was above 3 °C. In conclusion, temperature and humidity have a greater impact on the COVID-19 spread for most regions. However, the direction and intensity of their impact on the COVID-19 spread may be different due to differences in research objects or regional scales. In addition to temperature and humidity factors, rainfall, wind speed and evaporation also influence the epidemic spread (Table 1).

Although meteorological factors can accelerate or slow the speed of COVID-19 spread, scholars quickly realized that they were not the decisive factors. Therefore, some researchers tried to introduce atmospheric pollutant variables to explore the interactive influence of meteorological conditions and air quality on the epidemic spread, so as to accurately describe the driving mechanism of natural environmental factors on the spatial spread of the epidemic [35]. Compared with the studies on the impact of meteorological conditions on the epidemic spread, the differences are mainly manifested in three aspects: regional scale, index selection and research methods. In terms of the regional scale, studies on the impact of meteorological conditions and air quality on epidemic transmission focus on the urban scale because current air quality monitoring data are mainly carried out on the urban scale; the selected case cities include Seoul, New York, India, Wuhan, Xiaogan and Huanggang in China [36,37,38,39,40]. Most of these studies confirmed that PM_2.5_, PM_10_ and CO also had an impact on the epidemic spread; temperature, humidity and wind speed did not have an effect. However, different air pollutants had different directions and intensities on the epidemic spread due to the differences in case areas. In terms of index selection, major atmospheric pollutants such as CO, NO_2_, O_3_, PM_10_ and PM_2.5_ or comprehensive atmospheric pollutants such as AQI should be included in the evaluation index system for the impact of meteorological conditions and air quality on epidemic spread except for selecting factors such as temperature, humidity, wind speed, atmospheric pressure, sunshine, rainfall and evaporation. In terms of research methods, in addition to traditional correlation analysis, multiple regression analysis and the GAM model, the Poisson regression model, GLM model and GAMMs model have also been applied, which improves the accuracy of evaluation results.

### 2.2. Impact of Economic and Social Factors on the Epidemic Spread 

The spatial spread of COVID-19 has formed a specific geospatial pattern through changes in quantity, scale and spatio-temporal pattern, which to some extent reflects the spatial organization structure of human activities from the perspective of “place space” and “flow space” [16]. In the process of epidemic spread, people are carriers and transportation networks are channels, while economic and social linkages are the internal driving forces [21]. From this perspective, natural environmental factors are the external factors affecting the COVID-19 spread, while economic and social factors are the main factors. Overall, current studies focus on the impact of pure economic and social factors on the epidemic spread and the interaction between natural environment and economic and social factors on the epidemic spread.

In terms of pure economic and social factors, scholars mainly use qualitative and quantitative analysis methods to carry out some research (Table 2). In terms of qualitative analysis (the qualitative research method refers to the method that researchers use in historical review, literature analysis, interview, observation, participation experience and other methods to obtain research data, as well as in non-quantitative means to analyze them and obtain research conclusions), Zhang [41] analyzed the differences between cases in the whole of the United States, major cities in the United States, cities and suburbs, and communities within cities, and believed that the primary factor affecting the epidemic spread was workplace rather than population density and living conditions. De et al. [42] concluded that crowded living conditions, shared water and sanitation services, high dependence on public health services, limited communication tools and dependence on public transportation are the reasons for the spatial spread of the epidemic by mapping the case distribution in Gauteng, South Africa. Kuchler et al. [43] analyzed the association between confirmed cases and Facebook friends of two administrative departments in Westchester, and Lodi had connections with other cities; they concluded that the social connectivity index influenced the epidemic spread. Liu et al. [44] analyzed the information of confirmed cases in Zhuhai and believed that the inflow population of Wuhan and family gathering were the main cause of the epidemic spread. Zhao et al. [45] used big data mining technology to trace the spread trend of the epidemic in China and believed that the population exported from Wuhan had the greatest impact on the epidemic spread. The quantitative analysis methods (the quantitative research method is a research method and process that expresses problems and phenomena by quantity, and then analyzes and explains them, so as to obtain significance) are divided into traditional and spatial econometric analysis methods. In terms of traditional measurement methods, Chen et al. [46] used the Bayesian model to explore the relationship between epidemic transmission and population inflow in Wuhan and obtained a correlation coefficient of 0.943. Xiang et al. [47] used correlation analysis to explore the relationship between epidemic spread and population outflow in 31 provinces of China and found a positive hierarchical correlation between epidemic spread and population outflow. Wang et al. [48] used the Spearman correlation coefficient to find the relationship between epidemic transmission and the Baidu migration index in Guangdong Province and found that daily incidence was positively correlated with the 3-day migration index. Lin et al. [49] used multiple regression analysis to explore the influencing factors of epidemic transmission in 39 cities of China and believed that the number of tourists from Wuhan and the floating population in urban and rural areas were the key factors affecting epidemic transmission. Kraemer et al. [50] used the GLM model to illustrate the role of case importation in epidemic transmission, and also found that epidemic transmission was correlated with population movement. Sihaloho et al. [51] explored the impact of economic and social factors on the epidemic spread in Indonesia by means of multiple regression analysis, and revealed that the increase in the number of poor people, hotel room occupancy rate, urban Gini coefficient, population density, percentage of urban slum residents and percentage of household water use frequency for other purposes would promote the epidemic spread. Qiu et al. [52] used Ordinary Least Squares (OLS) to evaluate the impact of isolation policy, population flow, population density, city level and number of doctors on the epidemic spread in China, revealing that the prevention and control measures taken by the government and the number of people from the epidemic source are the key factors affecting the epidemic spread. Bassino and Ladmiral [53] used the two-layer change intercept model to explore the factors influencing the epidemic spread in Japan and believed that in addition to population density and international travel, residents’ income also affected the epidemic spread. In terms of spatial measurement methods, Gibson and Rush [54] used GIS spatial analysis to explore the influencing factors of epidemic transmission in Cape Town, South Africa, and believed that the existence of informal settlements would promote epidemic transmission. Famiglietti and Leibovici [55] used a vector autoregressive model to assess the role of health and economic policies in containing the epidemic spread in the United States, confirming the effectiveness of government interventions. Zheng and Liu [56] explored the influence of the built environment on the epidemic spread in Wuhan by means of the OLS method and geographically weighted regression model, and found that the source of infection, the degree of population concentration, the building density and the floor area ratio in the community all affected the epidemic spread.

In terms of the interaction between natural environment and economic and social factors, scholars mainly use correlation analysis, linear regression, nonlinear regression and spatial econometric analysis to carry out research. In terms of correlation analysis, Ahmadi et al. [57] used partial correlation coefficient and global sensitivity analysis to explore the impact of population density, population flow within the province, temperature, humidity, precipitation, wind speed and solar radiation on the epidemic spread in Iran, and thought that population density, population flow within the province, wind speed, humidity and solar radiation had an impact on the epidemic spread. Wang et al. [58] used the Spearman correlation coefficient and multiple regression analysis to explore the influencing factors of epidemic spread in 337 cities in China and found that the daily average temperature, wind speed, precipitation and strict index of government response were negatively correlated with epidemic spread, while air pollution and population migration were positively correlated with epidemic spread. In terms of linear regression, Dilek and Ali [59] used multiple regression analysis to explore the influence of daily maximum temperature, sunshine duration, population density, humidity, household income, population age 65 and above, proportion of African-Americans, Hispanics and whites on the COVID-19 spread in the United States, and revealed that population density and the proportion of African-Americans were key factors influencing the epidemic spread. Liu et al. [60] used multiple regression analysis to explore the influencing factors of epidemic spread in Shenzhen and found that the increase in GDP, actual foreign investment utilization and population density would promote epidemic spread. In terms of nonlinear regression, Le et al. [61] used the Clausius–Clapeyron regression equation to explore the impact of temperature, humidity and medical facilities (number of critically ill beds) on the epidemic spread in countries around the world and states in the United States, and found that for states in the United States, only medical facilities had an impact on the epidemic spread. For countries, the above factors influence the epidemic spread. Liu et al. [62] used the nonlinear regression method to explore the impact of meteorological conditions and population migration on the epidemic spread in China, and found that weather conditions with lower temperature, less variation in daytime temperature and lower humidity were more conducive to the epidemic spread. Wei et al. [63] used Logistic regression, GAM and the layered linear mixed model to explore the impact of transportation and meteorological factors on the epidemic spread in China, and revealed that low temperature, moderate cumulative precipitation, high wind speed and a large number of travelers would promote the epidemic spread. In terms of econometric, Baum and Henry [64] explored the impact of gender, race, age, income status, air pollutants and medical facilities on the epidemic spread in the United States and found that the above factors affected the epidemic spread. Besides, the epidemic spread was also affected by the spatial spillover effect of neighboring areas. Mollalo et al. [65] explored the impact of economic society (income, inequality, unemployment, etc.), environment (road network density, air quality, temperature, precipitation), topography (terrain, altitude, slope) and population (population over 65 years old, race, ethnicity, etc.) by using spatial error, spatial lag, geo-weighted regression (GWR) and the multi-scale geo-weighted regression model (MGWR), and found that income inequality, household income, the proportion of black women and nurse practitioners influence the epidemic spread.

## 3. Research Methods

### 3.1. Data Source and Processing Method

Information on COVID-19 cases (confirmed, cured, and dead), natural environment, and economic and social data are prerequisites for research on factors influencing the COVID-19 spread. At present, case information mainly comes from the following channels: First, the epidemic notifications issued by national or regional health departments are usually authoritative and mainly used epidemic research on different scales. However, for some economic underdeveloped countries or regions, there are often some problems in the epidemic notification, such as the lack of monitoring data in some regions, the imperfect monitoring information, the poor timeliness of data update, and the uneven information release channels, which will lead to misjudgment of the epidemic situation, and then affect the accuracy of the evaluation results of influencing factors of epidemic transmission. Second, epidemic data are released by research institutions or commercial institutions, such as the Global Pandemic Real-time Monitoring system (https://github.com/CSSEGISandData/COVID-19) developed by Johns Hopkins University, Live COVID-19 map of North America (https://coronavirus.1poCOVID int3acres.com/en) provided by 1Point3Acres, Live updates on COVID-19 (https://ncov.dxy.cn/ncovh5/view/pneumonia) provided by the Lilac Doctor App and real-time updates on COVID-19 provided by Tencent news App, etc. These types of data have been reproduced and quoted by many authoritative institutions at home and abroad because the data are reliable and time-sensitive, which is applicable to global, national, regional and urban macro and mesoscale research, but has limited application in micro-regional-scale research. The third is the epidemic data obtained by individuals or groups with the help of web crawler technology, which is mostly processed and sorted out on the basis of the above two epidemic data channels, and is often used to reveal the distribution law, evolutionary trend and driving mechanism of COVID-19 on the micro-regional scale. It should be noted that the acquisition of such data is subject to human interference, so the overall reliability of the data is lower than the epidemic data obtained through the previous two ways.

Natural environment and economic and social data mainly come from the following aspects: One is the data provided by monitoring stations, such as temperature, humidity, wind speed, sunshine, pressure and rainfall data provided by meteorological monitoring stations, and air pollutants data provided by air quality monitoring stations. The advantages of data are wide coverage, fast update speed, high accuracy and strong authority, while the disadvantages include being limited by the number of monitoring sites, which leads to the deviation of evaluation results when point source data are replaced by point source data. The second is relevant statistics released by government departments, such as resident income level, population size, population density, GDP, medical and health facilities, etc. Most scholars use economic and social data before 2020 as a substitute for epidemic research, which solves the problem of missing data to some extent, but also restricts the accuracy of evaluation results because COVID-19 research is concentrated in 2020 and there is a lag in the release of statistics. The third is the data obtained with the help of big data mining methods and technologies, such as mobile phone signaling data, Baidu population migration data, taxi track data, shared bike data, bus card swiping data, network social data, POI data, etc. The advantages of such data are the large amount of data and strong timeliness, which can better explain the causes of epidemic spread on the micro-regional scale. The disadvantages are that the data processing is difficult and the workload is large, which makes it difficult to reveal the deep-seated causes of epidemic spread on the macro-regional scale.

### 3.2. Main Research Methods

**Figure 1:**
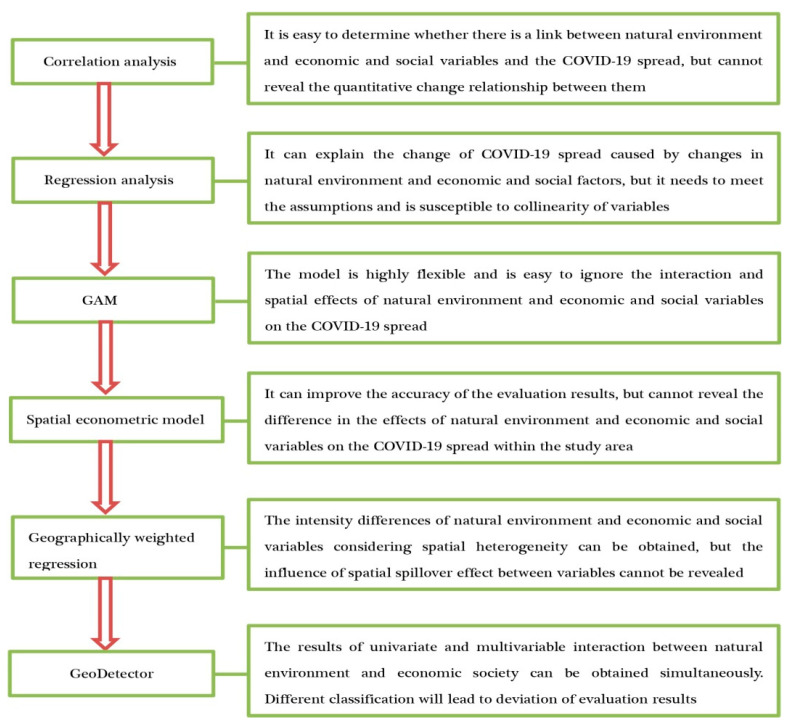
Main methods to study factors influencing the COVID-19 spread.

Quantitative analysis is the main method used to study the factors influencing the spatial spread of COVID-19, while the application of qualitative analysis is relatively limited. In the early stage of the outbreak, scholars mainly used correlation analysis methods such as the Pearson correlation coefficient, Spearman correlation coefficient and partial correlation coefficient to explore the impact of temperature, humidity, wind speed, air pressure, sunshine, rainfall, CO, NO_2_, O_3_, PM_10_, PM_2.5_, population density, population mobility, economic development level, medical facilities and urban built environment on the epidemic spread from the perspective of individual natural environment and economic and social factors. A large number of studies have confirmed the correlation between the above factors and epidemic spread. Although the correlation analysis method can reveal the relationship between some factors and epidemic spread, it cannot obtain the change of epidemic spread caused by the change of natural environment and economic and social factors. Therefore, some scholars tried to reveal the dose–response relationship between explanatory variables and explained variables by means of linear regression analysis methods (multiple linear regression, two-level intercept model and piecewise regression model). As the number of studies on COVID-19 continues to increase, some scholars believe that the relationship between natural environment and economic and social factors and the COVID-19 spread is not linear, so they try to quantify the relationship between them by using some nonlinear regression models (GAM, GLM and GAMMS). Both the traditional linear regression analysis and nonlinear regression analysis methods ignore the possible spatial position interdependence between the units in the study area, and the existence of spatial dependence will cause the deviation of the evaluation results obtained by the traditional regression model. In view of this, some scholars tried to reveal the impact of spatial dependence, spatial heterogeneity or interaction among factors on the epidemic spread rate by means of the spatial econometric model, geographically weighted regression model or geographic detector [66], which greatly improved the accuracy of model evaluation results (Figure 1). At the same time, as people’s understanding of the spatio-temporal evolution and spread rule of COVID-19 continues to deepen, some scholars have used qualitative analysis methods such as observation, comparison and induction and deduction to summarize the factors influencing the COVID-19 spread, which to a large extent helps to solve some problems that certain indicators have difficulty quantifying and promotes the research on the influencing factors of epidemic spatial spread to a deeper direction in theory and method.

## 4. Discussion

The paper systematically summarizes the factors influencing the spatial spread of COVID-19 from a geographical perspective and obtains a series of beneficial enlightenment, which can provide a reference value for formulating epidemic prevention and control measures. However, there are still uncertainties in this research in the following aspects due to the limitations of the researchers’ own technical means and cognitive level. First of all, the study mainly collects relevant basic literature materials from the Web of Science database and CNKI (China National Knowledge Infrastructure), and some important studies included in other literature databases may be omitted, which will result in incomplete review results of this study. In addition, this study adopted the method of combining subject retrieval and manual interpretation to screen the basic literature data, without considering the representativeness, reliability and publication bias of the literature. Although this method simplified the analysis process of this study, it also caused the uncertainty of the evaluation results. Secondly, the current research on factors influencing the spatial spread of COVID-19 in the field of geography focuses on revealing the spread process and mechanism of COVID-19, and the key factors influencing the evolution trend of COVID-19 are not explored enough, which weakens the application value of geographical research results to a large extent. In addition, the study of factors affecting the epidemic spread has been divided among different disciplines, and the comprehensive characteristics and interdisciplinary characteristics of geography have not been fully brought into play, which severely restricts the promotion of the status of geography, which also increases the uncertainty of the evaluation results. Thirdly, the literature collected in the study mainly focused on the factors influencing the spatial spread of COVID-19 in the early stage of the outbreak. As the mutation rate and types of COVID-19 strains continue to increase, the risk of epidemic spread and the difficulty of prevention and control are simultaneously increasing. At the same time, the factors affecting the COVID-19 spread also show complexity, dynamic and interactive characteristics, which virtually reduced the pertinence and persuasiveness of the evaluation results of the study. Finally, many countries and people have gradually realized that the COVID-19 epidemic will not end in the short term but will exist for a long time under the background of increasing downward pressure on the global economy. Therefore, they have changed their previous attitude of “zero tolerance” towards the COVID-19 virus and are prepared to live with it for a long time. The changes in the government’s and the public’s cognitive level will be reflected in the government’s control measures and the public’s behavioral preferences, which can affect the spatial spread of COVID-19. From this perspective, the factors influencing the COVID-19 spread in the new situation are obviously uncertain.

## 5. Conclusions

The factors influencing the spatial spread of COVID-19 have attracted more and more attention from the academic community, and the research content and methods have been further developed, providing a reference for further revealing the driving mechanism of the spread of COVID-19. However, in general, the following aspects need to be further deepened:

Research contents: (1) Selecting the evaluation index of influencing factors is not comprehensive enough. At present, the discussion on influencing factors of the spatial spread of COVID-19 is focused on meteorological factors, air quality and some economic and social factors. The influences of urban built environment, individual physiological differences, residents’ behavioral preferences and group psychological changes are ignored, and the role of these factors in the process of epidemic transmission needs to be further explored. (2) The mechanism of the epidemic spread has not been thoroughly discussed. In what ways does the natural environment and economic and social factors influence the spread speed of an epidemic? The relationship between human activities, geographical background and epidemic spread speed needs to be further explored in theory and model in a comprehensive and systematic way in the future. (3) Studies on the influencing factors of COVID-19 spread are mainly conducted on global, national, regional and urban scales, while micro studies at the level of streets, communities and residential areas are relatively scarce. As is known to all, streets, communities and residential areas are the basic components of a city, and there are differences in spatial structure, supporting facilities, economic level and population composition. In order to achieve the goal of precise prevention and control, it is imperative to carry out studies on the factors influencing the epidemic spread on the street, community and residential scales.

Research methods: (1) There are differences in data statistical caliber. The data used in the current study on factors influencing the COVID-19 spread mainly include daily resident case information, natural environment and some economic and social data (such as population migration and mobile phone signaling). However, the statistical data, such as resident income, gross domestic product (GDP) and medical and health conditions used are mainly annual data, and the difference in the statistical caliber of data will affect the accuracy of the evaluation results. (2) Some indicators are hard to quantify. When analyzing the impact of natural environment and economic and social factors on epidemic transmission, scholars mostly select indicators based on the principle of data accessibility and ignore the role played by indicators that are difficult to quantify (such as residents’ health, behavioral preference, cognitive level, psychological quality, public opinion and government intervention), which will also lead to incomplete evaluation results. (3) The difference of research methods leads to the unconvincing evaluation results. The research methods used to explore the influencing factors of epidemic transmission are complicated, and the differences in methods not only lead to differences in the evaluation results of influencing factors of epidemic transmission in different regions, but also lead to completely different evaluation results for the same region, which results in the lack of comparability between different research results. (4) The application of new technologies and methods is relatively limited. The causes affecting the epidemic spread are extremely complex, involving terrain, altitude, meteorological conditions, air quality, population density, economic development, medical facilities, physiological differences and behavioral preferences, etc. Besides, the interaction of factors has virtually intensified the difficulty of analysis. In addition, the interaction between the factors increases the difficulty of analysis. However, current scholars still analyze the driving mechanism of the epidemic spread mainly based on a number of limited variables with the help of traditional and spatial econometric analysis methods. Once the number of variables exceeds the limit, the stability of the model and the explanatory power of the conclusion will be reduced. Therefore, it is necessary to use systems thinking to introduce new technologies and methods into the study of the impact mechanism of epidemic spread in the future.

Geography can not only analyze the relationship between geographical background uncertainty and epidemic transmission from the perspective of natural environment, but also provide decision-making services for emergency management, social governance and public cognition under epidemic prevention and control from the perspective of humanistic economy, reflecting the responsibility of geography in serving national and local scientific decision making [67]. Although there are many studies on factors influencing the COVID-19 spread, the existing problems, such as indicator screening, data acquisition, scale effect and outdated methods, weaken the reference value of the evaluation results. It is suggested that future research should be strengthened in the following areas: (1) Attention should be paid to conducting a multi-scale, refined and systematic study on factors influencing the COVID-19 spread with the help of new technologies and methods. Existing studies on factors influencing the epidemic spread are mostly carried out on global, national, regional and urban scales, while studies at street, community and residential scales are relatively scarce, but they are crucial for the precise formulation of epidemic prevention and control measures. Especially in recent years, with the development of remote sensing, the geographic information system and big data mining technology, new technologies, new methods and new means continue to emerge, which makes it possible to conduct fine-grained, multi-scale and systematic studies on the factors influencing the epidemic spread in a specific geographical space because it is helpful to clarify the impact of scale effect on the evaluation results, thus profoundly revealing the driving mechanism of epidemic spread. (2) The focus should be on revealing the factors influencing the epidemic spread from the perspective of spatial and behavioral interaction. Human activities are an important factor affecting the COVID-19 spread. Therefore, future studies should focus on the spatial-temporal behavior and cognitive level of individual residents, supplemented by high-precision residents’ daily activity track and geographic information data, to deeply explore the interaction between human activities, geographic background and epidemic transmission, and provide reference for relevant departments to deal with emergent public health events. (3) Attention should be paid to the construction of residents’ case information, natural environment and economic and social basic databases. Complete information on residents’ cases, natural environment and economic and social data is the prerequisite for the analysis of influencing factors of epidemic transmission. Thus, attention should be paid to the construction of comprehensive database platforms in the future, to unify data caliber, broaden data acquisition, release and share channels, enrich and improve data mining methods, and enhance the scientificity and comparability of research results due to the serious lack of economic and social data used in current studies on factors influencing the COVID-19 spread.

## Figures and Tables

**Table 1:** Empirical study on the impact of natural environmental factors on the COVID-19 spread.

Research Method	Study Area	Research Conclusion	Typical Case
Niche model	Global	The lower the temperature, the faster the epidemic spreads	[24]
Spearman correlation coefficient	Four countries in South America	There is a negative correlation between humidity and incubation period	[27]
Generalized Additive Model (GAM)	50 counties in the United States	Higher temperatures, relative humidity and rainfall can reduce the risk of transmission	[32]
Pearson correlation coefficient	New Delhi, India	The daily maximum temperature, daily minimum temperature, daily mean temperature, relative humidity, evaporation and daily mean wind speed were positively correlated with the epidemic spread	[33]
Generalized Additive Mixed Models (GAMMs)	Chinese provincial units	Higher air pollutant concentration and lower temperature, relative humidity and wind speed are conducive to the epidemic spread	[35]
Generalized Linear Model (GLM)	5 megacities in India	PM_2.5_, PM_10_, CO, O_3_, Air Quality Index (AQI) and temperature affect the epidemic spread	[38]
Poisson regression model	Wuhan, Xiaogan and Huanggang in China	PM_2.5_ and humidity are positively correlated with epidemic spread, while PM_10_ and temperature are negatively correlated with epidemic spread	[39]

**Table 2:** Empirical study on the impact of economic and social factors on the COVID-19 spread.

Research Method	Study Area	Research Conclusion	Typical Case
Qualitative research method	Chinese prefecture-level units	Geographical proximity, population movement, population size, transportation network and epidemic prevention and control affect the spatial spread of the epidemic	[16]
	County-level unit of Henan Province	The epidemic spread is mainly affected by geographical proximity and population movement	[17]
Correlation analysis method	Two cities in the United States and Italy	The social connectedness index influences the epidemic spread	[43]
	Prefecture-level units in Guangdong Province	There is a positive correlation between the incidence of COVID-19 and the 3-day migration index	[48]
Linear regression method	Chongqing, China	Urban traffic factors, life service factors and street activity factors enhance the epidemic spread	[20]
	State units in United States	Population density and the proportion of AfricAn-Americans influence the epidemic spread	[59]
GLM	Wuhan, China	Population mobility affects the spatial spread of the epidemic	[50]
Clausius–Clapeyron regression equation	Countries around the world and American states	Medical facilities affect the epidemic spread in each state of the United States, while temperature, humidity and medical facilities affect the epidemic spread in each country	[61]
Logistic regression, GAM, hierarchical linear mixed model	County administrative unit in China	Low average temperature, moderate accumulated precipitation, high wind speed and large number of travellers have significant influence on the epidemic spread	[63]
Spatial econometric model	48 states and counties in the United States	Gender, race, age, income, air pollution and medical facilities affect the epidemic spread	[64]

## References

[1] Liu W. (2020). The impacts of COVID-19 pandemic on the development of economic globalization. Geogr Res.

[2] Valjarević A., Milić M., Valjarević D., Stanojević-Ristić Z., Petrović L., Milanović M., Filipović D., Ristanović B., Basarin B., Lukić T. (2020). Modelling and mapping of the COVID-19 trajectory and pandemic paths at global scale: A geographer’s perspective. Open Geosci..

[3] Wu F., Zhao S., Yu B., Chen Y., Wang W., Song Z., Hu Y., Tao Z., Tian J., Pei Y. (2020). A new coronavirus associated with human respiratory disease in China. Nature.

[4] David S.H., Esam I.A., Tariq A.M., Francine N., Richard K., Osman D., Giuseppe I., Timothy D.M., Zaid A.M., Christian D. (2020). The continuing 2019-nCoV epidemic threat of novel coronaviruses to global health—The latest 2019 novel coronavirus outbreak in Wuhan, China. Int. J. Infect. Dis..

[5] Liu T., Hu J., Kang M., Lin L., Zhong H., Xiao J., He G., Song T., Huang Q., Rong Z. (2020). Transmission dynamics of 2019 novel coronavirus (2019-nCoV). BioRxiv.

[6] Fanelli D., Piazza F. (2020). Analysis and forecast of COVID-19 spreading in China, Italy and France. Chaos. Soliton. Fract..

[7] Zheng Z., Xie Z., Qin Y., Wang K., Fu P. (2021). Exploring the influence of human mobility factors and spread prediction on early COVID-19 in the USA. BMC. Public Health.

[8] Zhu Y., Xie J., Huang F., Cao L. (2020). Association between short-term exposure to air pollution and COVID-19 infection: Evidence from China. Sci. Total Environ..

[9] Ahmed J., Jaman M.H., Saha G., Ghosh P. (2021). Effect of environmental and socio-economic factors on the spreading of COVID-19 at 70 cities/provinces. Heliyon.

[10] Zhang K., Qian Q. (2020). The impact of COVID-19 on China’s economy and discussion of policies-evidence from listed Companies. Trop. Geogr.

[11] Du F., Wang J., Wang H. (2020). The impacts of COVID-19 on the connectivity of China’s international air transport network and the spatial differences. Trop. Geogr.

[12] Franch-Pardo I., Desjardins M.R., Barea-Navarro I., Cerdà A. (2021). A review of GIS methodologies to analyze the dynamics of COVID-19 in the second half of 2020. Trans. GIS.

[13] Zhao R. (2020). Introduction to Geography’s Relationship with the Epidemic Prevention and Control. http://blog.sciencenet.cn/blog-3155012-1220355.html.

[14] Chai Y., Xu W., Zhang W., Li C., Li Y. (2020). A research framework of precise epidemic prevention and control from the perspective of Space-time Behavioral Geography. Sci. Geogr. Sin..

[15] Franch-Pardo I., Napoletano B.M., Rosete-Verges F., Billa L. (2020). Spatial analysis and GIS in the study of COVID-19. A review. Sci. Total. Environ..

[16] Wang J., Du D., Wei Y., Yang H. (2020). The development of COVID-19 in China: Spatial diffusion and geographical pattern. Geogr. Res..

[17] Liu Y., Yang D., Dong G., Zhao H., Miao C. (2020). The spatio-temporal spread characteristics of 2019 Novel Coronavirus Pneumonia and risk assessment based on population movement in Henan Province: Analysis of 1243 individual case reports. Econ. Geogr..

[18] Fu B., Zhang J., Wang S., Zhao W. (2020). Classification-coordination-collaboration: A systems approach for advancing sustainable development goals. Natl. Sci. Rev..

[19] Kumar R., Pandey A., Ibsa R.G., Sinwar D., Dhaka V.S. (2021). Study of social and geographical factors affecting the spread of COVID-19 in Ethiopia. J. Stat. Manag. Syst..

[20] Chen X., Huang Y., Li J., Wang S., Pei T. (2020). Clustering characteristics of COVID-19 cases and influencing factors in Chongqing Municipality. Prog. Phys. Geog..

[21] Xie Z., Qin Y., Li Y., Shen W., Zheng Z., Liu S. (2020). Spatial and temporal differentiation of COVID-19 epidemic spread in mainland China and its influencing factors. Sci. Total Environ..

[22] Cai Y. (2019). Integrated Physical Geography.

[23] Walrand S. (2020). Autumn COVID-19 surge dates in Europe correlated to latitudes, not to temperature-humidity, pointing to vitamin D as contributing factor. Sci. Rep..

[24] Araújo M.B., Naimi B. (2020). Spread of SARS-CoV-2 Coronavirus likely to be constrained by climaters. MedRxiv.

[25] Bannister-Tyrrell M., Meyer A., Faverjon C., Cameron A. (2020). Preliminary evidence that higher temperatures are associated with lower incidence of COVID-19, for cases reported globally up to 29th February 2020. MedRxiv.

[26] Bariotakis M., Sourvinos G., Castanas E., Pirintsos S.A. (2020). Climatic influences on the worldwide spread of SARS-CoV-2. MedRxiv.

[27] Zhu L., Liu X., Huang H., Ricardo A.L., Mauricio M.L.L., Aldo G., Ricardo S.R., Leandro P., Magaly V.A., Benoit D. (2020). Meteorological impact on the COVID-19 pandemic: A study across eight severely affected regions in South America. Sci. Total Environ..

[28] Tosepu R., Gunawan J., Effendy D.S., Ahmad L., Asfian P. (2020). Correlation between weather and Covid-19 pandemic in Jakarta, Indonesia. Sci. Total Environ..

[29] Gupta S., Raghuwanshi G.S., Chanda A. (2020). Effect of weather on COVID-19 spread in the US: A prediction model for India in 2020. Sci. Total Environ..

[30] Qi H., Xiao S., Shi R., Ward M.P., Chen Y., Tu W., Su Q., Wang W., Wang X., Zhang Z. (2020). COVID-19 transmission in Mainland China is associated with temperature and humidity: A time-series analysis. Sci. Total Environ..

[31] Gupta D., Gupta A. (2020). Effect of Ambient Temperature on COVID 19 Infection rate: Evidence from California. SSRN.

[32] Chien L.C., Chen L.W. (2020). Meteorological impacts on the incidence of COVID-19 in the U.S. Stoch. Environ. Res. Risk A.

[33] Singh O., Bhardwaj P., Kumar D. (2020). Association between climatic variables and COVID-19 pandemic in National Capital Territory of Delhi, India. Environ. Dev. Sustain..

[34] Xie J., Zhu Y. (2020). Association between ambient temperature and COVID-19 infection in 122 cities from China. Sci. Total Environ..

[35] Cao H., Li B., Gu T., Liu X. (2020). Associations of ambient air pollutants and meteorological factors with COVID-19 transmission in 31 Chinese provinces: A time-series study. medRxiv.

[36] Yong K.L., Kweon O.J., Kim H.R., Kim T.H., Lee M.K. (2021). The impact of environmental variables on the spread of COVID-19 in the Republic of Korea. Sci. Rep..

[37] Bashir M.F., Ma B., Komal B., Komal B., Bashir M. (2020). Correlation between climate indicators and COVID-19 pandemic in New York. Sci. Total Environ..

[38] Kolluru S., Patra A.K., Allaudeen N., Nagendra S.S. (2021). Association of air pollution and meteorological variables with COVID-19 incidence: Evidence from five megacities in India. Environ. Res..

[39] Jiang Y., Wu X.J., Guan Y.J. (2020). Effect of ambient air pollutants and meteorological variables on COVID-19 incidence. Infect. Control Hosp. Epidemiol..

[40] Zhang Z., Xue T., Jin X. (2020). Effects of meteorological conditions and air pollution on COVID-19 transmission: Evidence from 219 Chinese cities. Sci. Total Environ..

[41] Zhang T. (2020). Impacts of the built environment on the COVID-19 epidemic and the evidence-based practice: A preliminary analysis of the COVID-19 epidemic in American cities. City Plan. Rev..

[42] Brailovskaia J., Cosci F., Mansueto G., Margraf J. (2020). The relationship between social media use, stress symptoms and burden caused by coronavirus (Covid-19) in Germany and Italy: A cross-sectional and longitudinal investigation. J. Affect. Disord. Rep..

[43] Kuchler T., Russel D., Stroebel J. (2021). The geographic spread of COVID-19 correlates with the structure of social networks as measured by Facebook. J. Urban Econ..

[44] Liu Z., Ye Y., Zhang H., Guo H., Yang J., Wang C. (2020). Spatio-temporal characteristics and transmission path of COVID-19 cluster cases in Zhuhai. Trop. Geogr..

[45] Zhao X., Li X., Nie C. (2020). Backtracking transmission of COVID-19 in China based on big data source, and effect of strict pandemic control policy. Bull. Chin. Acad. Sci..

[46] Chen Z., Zhang Q., Lu Y., Guo Z., Zhang X., Zhang W., Guo C., Liao C., Li Q., Han X. (2020). Distribution of the COVID-19 epidemic and correlation with population from Wuhan, China. Chin. Med. J..

[47] Xiang Y., Wang S. (2020). Spatial relationship between epidemic spread and population outflow of the Corona Virus Disease 2019 (COVID-19) that impacted Chinese urban public health classification. Trop. Geogr..

[48] Wang X., Liao C., Li Z., Hu H., Cheng X., Li Q., Lu J. (2020). Preliminary analysis on the early epidemic and spatiotemporal distribution of new coronavirus pneumonia in Guangdong Province. J. Trop. Med..

[49] Lin Y., Zhong P., Chen T. (2020). Association between socioeconomic factors and the COVID-19 outbreak in the 39 well-developed cities of China. Front. Public Health..

[50] Kraemer M., Yang C.H., Gutierrez B., Wu C., Klein B., Plgott D.M., Plessis L.D., Faria N.R., Li R., Hanage W. (2020). The effect of human mobility and control measures on the COVID-19 epidemic in China. Science.

[51] Sihaloho E.D., Dana W., Siregar C. (2020). Impacts of regional economic factors on the transmission of coronavirus disease 2019 (COVID-19) in Indonesia. Ekon. J. Econ. Bus..

[52] Qiu Y., Chen X., Shi W. (2020). Impacts of social and economic factors on the transmission of Coronavirus Disease 2019 (COVID-19) in China. J. Popul. Econ..

[53] Bassino J.P., Ladmiral G. (2020). Socio-economic factors influencing COVID-19 spread in Japan evidence from the second wave. SSRN Electron. J..

[54] Gibson L., Rush D. (2020). Novel coronavirus in Cape Town informal settlements: Feasibility of using informal dwelling outlines to identify high risk areas for COVID-19 transmission from a social distancing perspective. JMIR Public Health Surveill..

[55] Famiglietti M., Leibovici F. (2021). The impact of health and economic policies on the spread of COVID-19 and economic activity. Work. Pap..

[56] Zheng T., Liu H. (2020). Exploration of the built-environmental elements that influence the spread of COVID-19 pandemic on community scale: A case study of Wuhan, China. Mod. Urban. Res..

[57] Ahmadi M., Sharifi A., Dorosti S., Ghoushchi S.J., Ghanbarl N. (2020). Investigation of effective climatology parameters on COVID-19 outbreak in Iran. Sci. Total Environ..

[58] Wang Q., Dong W., Yang K., Ren Z., Wang J. (2021). Temporal and spatial analysis of COVID-19 transmission in China and its influencing factors. Int. J. Infect. Dis..

[59] Dilek M., Ali M. (2020). Analysis of environmental, economic, and demographic factors affecting COVID-19 transmission and associated deaths in the U.S.A. SSRN Electron. J..

[60] Liu S., Qin Y., Xie Z., Zhang J. (2020). The spatial-temporal characteristics and influenciing factors of COVID-19 spread in Shenzhen, China—An analysis based on 417 cases. Int. J. Environ. Res. Public Health.

[61] Le N.K., Le A.V., Parikh J.U., Brooks J., Gardellini T., Lzurieta R. (2021). Ecological and Health infrastructure factors affecting the transmission of mortality of COVID-19. J. Adv. Virol. Res..

[62] Liu J., Zhou J., Yao J., Zhang X., Zhang K. (2020). Impact of meteorological factors on the COVID-19 transmission: A multi-city study in China. Sci. Total Environ..

[63] Wei J.T., Liu Y.X., Zhu Y.C., Qian J., Cao W.C. (2020). Impacts of transportation and meteorological factors on the transmission of COVID-19. Int. J. Hygrogen Environ. Health.

[64] Baum C.F., Henry M. (2020). Socioeconomic factors influencing the spatial spread of COVID-19 in the United States. SSRN Electron. J..

[65] Mollalo A., Vahedi B., Rivera K.M. (2020). GIS-based spatial modeling of COVID-19 incidence rate in the continental United States. Sci. Total Environ..

[66] Cao Y., Liu Y., Zhou C. (2021). Spatiotemporal diffusion characteristics and influencing factors of COVID-19 epidemic from the perspective of urban agglomeration. Areal. Res. Dev..

[67] Xue B., Xiao X., Su F., Tang C., Cheng Y., Xie X., Zhao H., Wang Y., Zhang Z., Li J. (2020). Geographical academic responses and outlook in the novel coronavirus pneumonia epidemic prevention and control. Sci. Geogr. Sin..

